# Applications of LIF to Document Natural Variability of Chlorophyll Content and Cu Uptake in Moss

**DOI:** 10.3390/plants13152031

**Published:** 2024-07-24

**Authors:** Kelly Truax, Henrietta Dulai, Anupam Misra, Wendy Kuhne, Celia Smith, Ciara Bongolan-Aquino

**Affiliations:** 1 Department of Earth Sciences, University of Hawai‘i at Mānoa, Honolulu, HI 96822, USA; hdulaiov@hawaii.edu (H.D.); anupam@hawaii.edu (A.M.); caquino6@hawaii.edu (C.B.-A.); 2 Savannah River National Laboratory, Aiken, SC 29831, USA; wendy.kuhne@srnl.doe.gov; 3 School of Life Science, University of Hawai‘i at Mānoa, Honolulu, HI 96822, USA; celia@hawaii.edu

**Keywords:** bryophyte, metal contamination, environmental stress, metal detection, laser-induced fluorescence, image processing

## Abstract

Chlorophyll has long been used as a natural indicator of plant health and photosynthetic efficiency. Laser-induced fluorescence (LIF) is an emerging technique for understanding broad spectrum organic processes and has more recently been used to monitor chlorophyll response in plants. Previous work has focused on developing a LIF technique for imaging moss mats to identify metal contamination with the current focus shifting toward application to moss fronds and aiding sample collection for chemical analysis. Two laser systems (CoCoBi a Nd:YGa pulsed laser system and Chl-SL with two blue continuous semiconductor diodes) were used to collect images of moss fronds exposed to increasing levels of Cu (1, 10, and 100 nmol/cm^2^) using a CMOS camera. The best methods for the preprocessing of images were conducted before the analysis of fluorescence signatures were compared to a control. The Chl-SL system performed better than the CoCoBi, with dynamic time warping (DTW) proving the most effective for image analysis. Manual thresholding to remove lower decimal code values improved the data distributions and proved whether using one or two fronds in an image was more advantageous. A higher DTW difference from the control correlated to lower chlorophyll a/b ratios and a higher metal content, indicating that LIF, with the aid of image processing, can be an effective technique for identifying Cu contamination shortly after an event.

## 1. Introduction

Bryophytes have long been used as bioindicators of heavy metal accumulation in the environment due to various anthropogenic sources [1,2]. Of the species commonly used, mosses are the most widely distributed across differing elevations and climates. Mosses have a simple non-vascular structure, lacking true roots, enabling them to accumulate most of their nutrients from the atmosphere [3,4]. Because they are often only one or two cell-layers thick, mosses have a high ion exchange affinity due to their lack of a protective epidermis, leading to the absorption of both inorganic and organic compounds [5,6]. When compared to vascular plants, mosses consistently prove more effective in metal accumulation [2]. The use of moss for environmental sampling has been validated through chemical analysis [7,8] and has proved to be valuable for large-scale surveys [9,10] by being capable of absorbing nutrients and contaminants directly from the atmosphere.

A good bioindicator is defined as an organism that can successfully provide new information about the quality of a given environment [11] while also being capable of monitoring potential pollutants and their temporal and spatial distributions [12]. To be an effective biomonitor, a plant species must be capable of detecting changes over time in the environment and help in source identification [13]. Because mosses can be found on every continent and over a vast array of ecosystems, they are uniquely capable of surviving in harsh and often highly polluted areas [14,15]. Mosses also benefit from being a species that can withstand repeated sampling with the potential to provide annual growth segments for the continuous monitoring of both recent and historical heavy metal accumulation [11,14,16,17].

However, the accumulation of heavy metal pollutants can occur through several different mechanisms, depending on the moss species and physiological mechanisms for ionic exchange. Some particulates can become trapped on the surface of cells due to negatively charged anionic sites, others are incorporated into cellular walls, while higher affinity metals are more likely to be used in metabolic processes within the cell itself [11,18]. Metal retention efficiency has been documented to decrease in the order of Cu > Pb > Ni > Zn, with Cu and Pb showing the strongest correlation between metal concentration and atmospheric deposition [16,19]. Pb’s toxicity, even in small doses, is well-known for its potential health concerns both to humans and the environment, but even an essential micronutrient like Cu can be harmful in high concentrations [20,21,22]. Because of its high affinity and need for use in metabolic processes, Cu provides a key example of metal behavior to best understand and document physiological response. Though mosses are more cost effective than atmospheric collectors and are able to accumulate wide ranges of heavy metals [2,23,24], the sampling and chemical analysis can be laborious and time consuming. We propose that environmental sampling could be expedited by the addition of remote sensing using laser-induced fluorescence (LIF).

The use of chlorophyll fluorescence to evaluate plant physiology and characterize photosynthetic efficiency has long been used to determine vascular structure and metabolic processes [25,26,27]. Lasers can be used as a primary source for inducing fluorescence in plants [27,28] to monitor the chlorophyll content and changes due to metal or environmental stress [29,30,31]. Chlorophyll is an important acceptor and emitter of visible light energy during photosynthetic processes, which allows LIF the opportunity to detect changes in chlorophyll through documented shifts in the short lifetime fluorescence emission of light. This shift corresponds to the absorption of the laser’s wavelength by a molecule and the spontaneous emission of light resulting from its return to its ground energy state [32,33,34,35]. It has been documented in the literature that metals can bind to chlorophyll and protein structures to produce specific fluorescence signatures [31,32,34,36]. Therefore, LIF shows promise of an in situ application for the repeated sampling of a given plant or area through non-destructive means.

Success using LIF has been demonstrated in previous work using the Color Compact Biofinder (CoCoBi) [37,38,39], which uses two nanosecond pulsed lasers fired in tandem (355 nm UV and 532 nm green) to detect a broad range of organic molecule responses using a CMOS camera. However, the low selectivity of that technique does not allow for the direct identification of the process responsible for the fluorescence response. Thus, a chlorophyll specific laser system (Chl-SL) was developed that focuses on chlorophyll absorption by using laser wavelengths of 445 nm and 462 nm (Chl-a and -b), which produce emissions in the visible red region (650 nm for chl-b and 670 nm for chl-a) [30] and can be documented with the same CMOS camera previously used with the CoCoBi [38]. The CoCoBi laser wavelengths are good for a broad range of organics, but are not specifically designed for chlorophyll, which has led to a lower sensitivity to metal contamination than desired. By developing the Chl-SL to focus on the maximum absorption wavelengths for chlorophyll, we can receive the maximum corresponding light emission, which should greatly improve the sensitivity to chlorophyll changes.

Previous work [39] has focused on the image analysis of moss mats using LIF. However, it was observed that the analysis of moss mats resulted in an averaged evaluation that was not representative of the natural variability found between individual fronds. It was determined that to evaluate whether a given frond selected from a moss mat was representative of contamination, it was necessary to ensure successful sampling for potential chemical analysis. In addition, as we wanted to better understand the role of chlorophyll and metal content on the LIF plant response, we decided to study individual fronds to better understand the underlying biological processes and determine the effectiveness of LIF of both the CoCoBi and Chl-SL on a smaller scale. As with any plant, moss fronds can have a high level of variation in chlorophyll content, and therefore in fluorescence response, even amongst healthy samples. To effectively evaluate and compare images collected using LIF, more traditional chemical analyses of chlorophyll and metal extraction will be used for validation. It is expected that the technique can help provide the identification of Cu contamination within moss samples and find a correlation to chlorophyll content and metal accumulation while creating a methodology that is reproducible. The ultimate goal is to provide a method of sampling individual fronds that are highly representative of a contaminated moss mat identified using LIF in the environment. We recognize that LIF is limited in its specificity compared to traditional destructive techniques of metal content analysis, but through this study, we demonstrate that LIF can help to find and sample target areas in a way that complements traditional bioassay approaches through targeted sampling at potential contaminated sites.

## 2. Results

### 2.1. Comparison of LIF Technique to Moss Fronds

**Figure 1:**
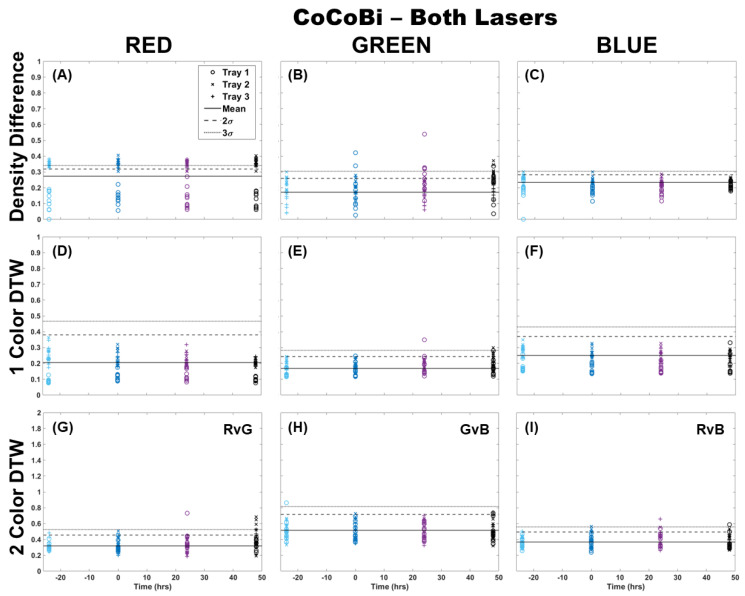
Comparison of moss response to both lasers of the CoCoBi using three image analysis methods: (**A**–**C**) single-color density difference, (**D**–**F**) single-color DTW, and (**G**–**I**) two-color DTW. Images of fronds were collected every 24 h over three days. At time 0, three Cu treatments were given at 1 nmol/cm^2^ for Tray 1 (o’s), 10 nmol/cm^2^ for Tray 2 (x’s), and 100 nmol/cm^2^ for Tray 3 (+’s).

The analysis of fronds was initially limited to comparing only those images from which chlorophyll was extracted (single fronds). Pairs of fronds imaged for metal extraction were withheld from this initial analysis for use in the validation of the technique at a later step. Fronds were imaged using both laser systems (CoCoBi and Chl-SL), with results for all color channels and all analytical methods shown for the CoCoBi in Figure 1. The CoCoBi consistently showed better results when using the green and UV laser in tandem. When considering the methods of analysis, two-color DTW showed no deviation from the control at any point during the experiment, save for one control sample for Tray 1. Both single-color analysis methods showed a deviation in the red and blue color channels 24 h after metal dosing for Tray 3, with no deviation in the green color channel. Of these methods, the density difference appeared to perform least well, with several samples from all three trays deviating from the 3σ confidence interval of the control when looking at the red color channel. The blue color channel only deviated in Tray 3 24 h after dosing. The single-color DTW was in good agreement, indicating that the CoCoBi may be measuring a metabolic response to the introduction of Cu 24 h after the initial dosing. However, when further inspecting images collected with the CoCoBi, the blue channel response appeared to be linked to a change in the tips of the fronds, which could indicate a growth response in the moss that is not specific to chlorophyll fluorescence.

**Figure 2:**
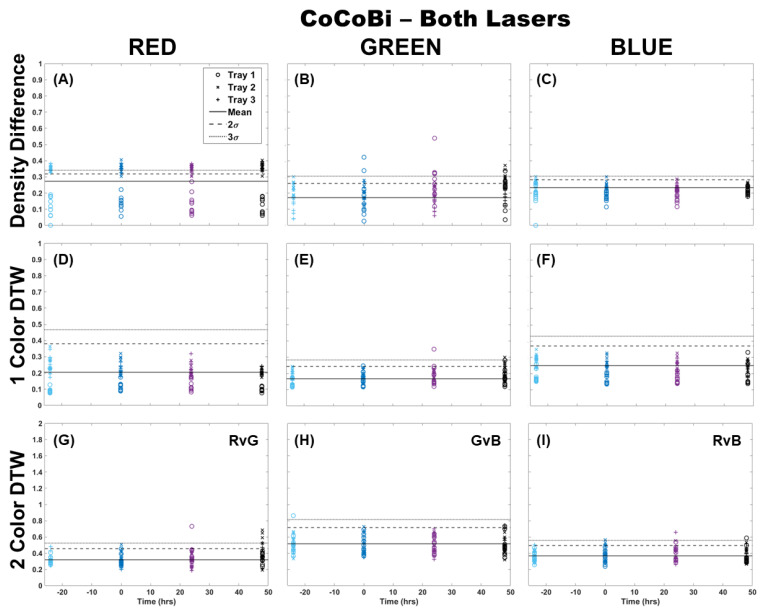
Comparison of moss response to the Chl-A laser (445 nm) with the Chl-B filter (650 nm) of the Chl-SL system using three image analysis methods: (**A**–**C**) single-color density difference, (**D**–**F**) single-color DTW, and (**G**–**I**) two-color DTW. Images of fronds were collected every 24 h over three days. At time 0, three Cu treatments were given at 1 nmol/cm^2^ for Tray 1 (o’s), 10 nmol/cm^2^ for Tray 2 (x’s), and 100 nmol/cm^2^ for Tray 3 (+’s).

When using the Chl-A laser, application of the Chl-B filter improved the image analysis results for all methods by providing better separation from the control samples and between trays (Figure 2). Again, it can be observed that the results using density difference varied between color channels and between trays as well as time since Cu dosing. The blue color channel was the most representative of the deviation expected of Cu dosing with clear separation using both the single-color and two-color analysis methods, with only the higher dosed Trays 2 and 3 separating from the control. There was almost no deviation in single-color DTW for the red and green color channels, but two-color DTW revealed good separation from the control at time 0, and some separation of individual fronds from Trays 2 and 3 at 24 h after dosing. The results here, however, are still muddled when considering the red channel results, especially for the two-color analysis of red versus green (RvG), where there was separation from the control by some samples before dosing occurred.

**Figure 3:**
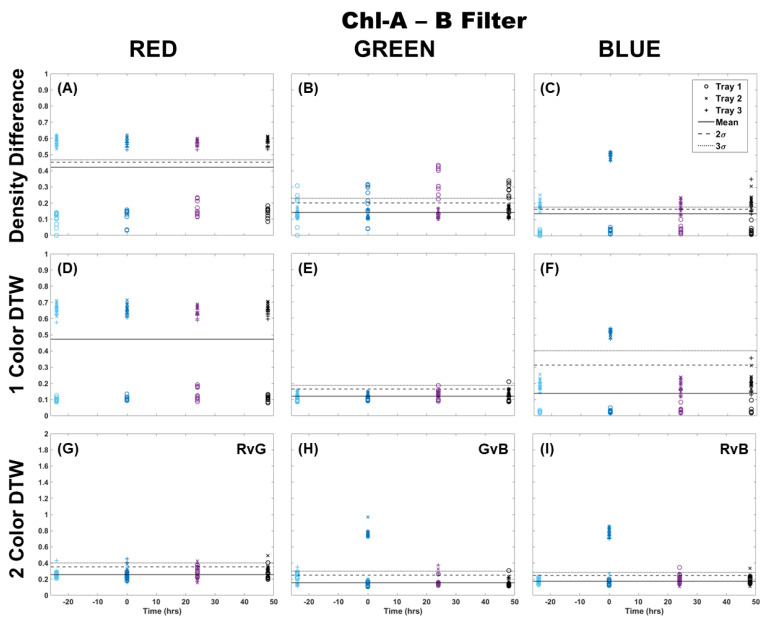
Comparison of moss response to the Chl-B laser (462 nm) with the Chl-B filter (650 nm) of the Chl-SL system using three image analysis methods: (**A**–**C**) single-color density difference, (**D**–**F**) single-color DTW, and (**G**–**I**) two-color DTW. Images of fronds were collected every 24 h over three days. At time 0, three Cu treatments were given at 1 nmol/cm^2^ for Tray 1 (o’s), 10 nmol/cm^2^ for Tray 2 (x’s), and 100 nmol/cm^2^ for Tray 3 (+’s).

The Chl-B laser with application of the Chl-B filter improved upon the results in Figure 2 regardless of the analysis method. It can be seen in Figure 3 that the density difference method still showed wide variability between the samples and separation from the control 3σ before and after Cu dosing. However, it did show a change in the same distribution of points when comparing the blue color channel to red and green. Again, single-color DTW performed better, but there were still a few samples that deviated from the control in each of the color channels. The separation at the time of dosing (time 0) was well represented in the blue color channel, while the red and green showed an outlier from the general population results. Two-color DTW showed good separation in both GvB and RvB for Trays 2 and 3. It also appeared to show separation between the two dosing levels in GvB, with the Tray 3 samples slightly higher than those of Tray 2. There were still a few responses 24 and 48 h later, but the initial Cu dosing was well-separated and distinguishable from these perhaps prolonged metabolic processes. Though quite similar, the Chl-B laser with Chl-B filter (Figure 3) may provide slightly better specificity to individual frond response than that observed with the Chl-A laser with Chl-B filter (Figure 2).

**Figure 4:**
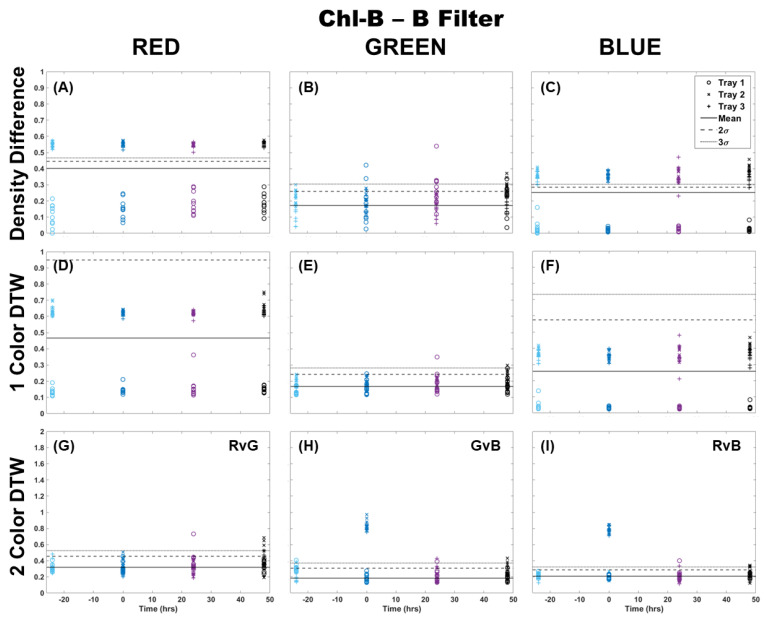
Comparison of three laser systems (CoCoBi—(**A**,**D**,**G**); Chl-A—(**B**,**E**,**H**); Chl-B—(**C**,**F**,**I**)) and three analysis methods (density difference—(**A**–**C**); single-color DTW—(**D**–**F**); two-color DTW—(**G**–**I**)) in the blue or RvB color channel. Images of fronds were collected every 24 h over three days. At time 0, three Cu treatments were given at 1 nmol/cm^2^ for Tray 1 (o’s), 10 nmol/cm^2^ for Tray 2 (x’s), and 100 nmol/cm^2^ for Tray 3 (+’s).

In Figure 4, all blue-color channel or two-color RvB results from each of the laser systems (Figure 1, Figure 2 and Figure 3) were compiled for direct comparison. The 24-h response after Cu dosing recorded by the CoCoBi was especially prominent here for Tray 3, but not well-documented using the chlorophyll specific lasers. The Chl-A and Chl-B results showed differing ranges for healthy chlorophyll response, but were in good agreement for separation at the time of Cu dosing (time 0). Upon closer inspection, Chl-A, regardless of analysis method, showed tightly condensed ranges for the Tray 2 and 3 results at time 0, while Chl-B showed better separation from the control 3σ for both the DTW analysis of single and two colors while also showing good separation between individual fronds. Regardless of laser, density difference performed poorly when compared to DTW, even if the overall distributions of points were similar. Because the Chl-B results had better separation between individual frond samples, its two-color DTW results were compared to chlorophyll and metal extraction.

### 2.2. Comparison of LIF Analysis of Chl-SL and SET Fronds

**Figure 5:**
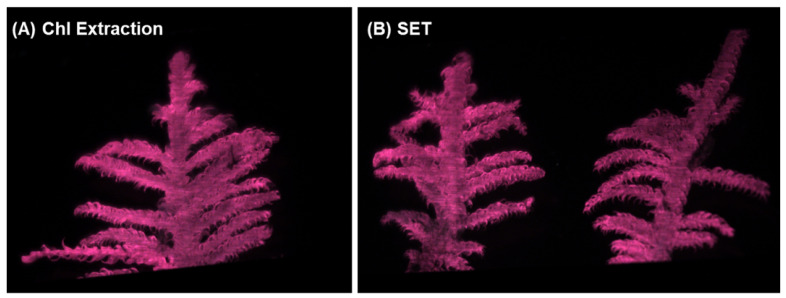
(**A**) LIF image collected of a single frond that later underwent chlorophyll extraction. (**B**) LIF image of a pair of fronds that underwent metal extraction.

**Figure 6:**
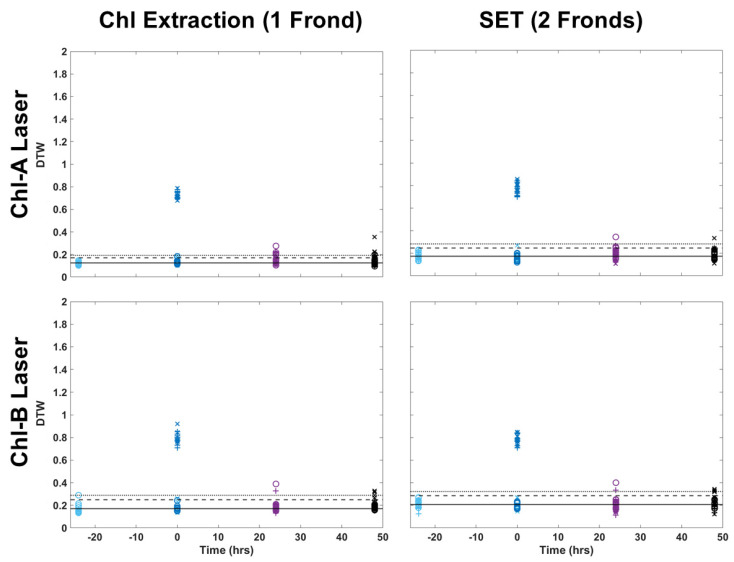
Comparison of two-color DTW RvB results for both the Chl-A and Chl-B lasers with the Chl-B filter when applied to single fronds from chlorophyll extraction and pairs of fronds collected for metal extraction (SET).

Two-color DTW was also applied to images collected of frond pairs for metal extraction. Figure 5 shows a visual comparison of how the images looked for a single frond collected for chlorophyll extraction and a pair of fronds collected for metal extraction. Figure 6 applied two-color DTW to images collected for both chemical analysis techniques for both the Chl-A and Chl-B lasers while using the Chl-B filter. There were subtle differences between the images of single and pairs of fronds, but overall, the distribution of samples was very similar, regardless of the laser or number of fronds within the image analyzed. Pairs of fronds may have shown less separation between individual samples when compared to single fronds, but their degree of difference using two-color DTW was in the same region of the plot. The range of the control was more similar between the Chl-A and Chl-B laser when observing frond pairs, but shifted minimally when comparing single versus pairs for the Chl-B laser. These results provide us with confidence that the technique of two-color DTW applied to images that have undergone thresholding of 10 DCV is repeatable for future samples as well as easily adaptable to samples of different shapes and sizes.

### 2.3. Chlorophyll Extraction and SET Results

**Figure 7:**
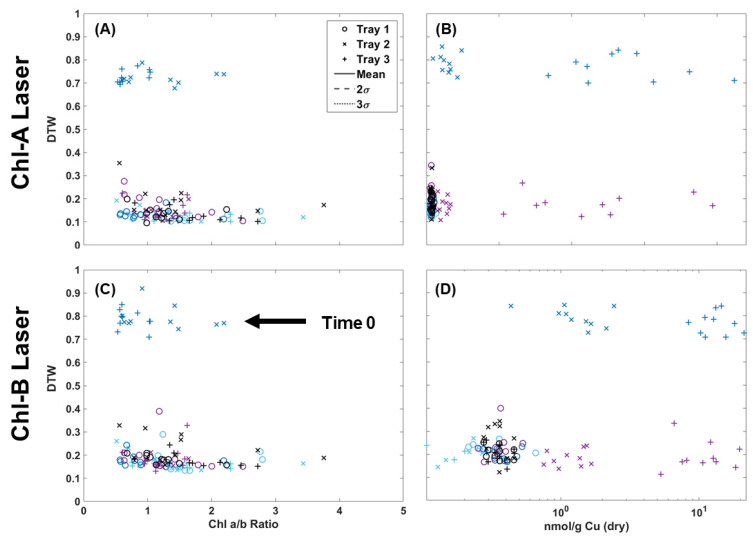
Chlorophyll extraction results of chl a/b ratio compared to the two-color DTW results from images collected using the Chl-A (**A**) and Chl-B (**C**) lasers using the B filter. Metal extraction results of Cu dry weight of fronds collected every 24 h compared to the Chl-A (**B**) and Chl-B (**D**) two-color DTW results.

Table 1 shows the results of chlorophyll extraction for single fronds collected over the 72 h of the experiment, while Table 2 shows the Cu concentrations for both the wet and dry weight. It should be noted that while the chlorophyll content was measured for all fronds, only five samples from each tray collected before Cu dosing (control) were sent for metal analysis via ICP-MS (15 in total). In Figure 7, the two-color DTW RvB results are plotted against the Chl a/b ratio for individual fronds and Cu dry weight values (nmol/g) for pairs of fronds. The chlorophyll results showed a more compacted distribution of points when using the Chl-A laser. Although the Chl-B data were sparser, there was still clear separation for the samples not collected on the same day of Cu dosing. Samples collected on non-dosing days all stayed below a 0.5 difference while Tray 2 and Tray 3 samples evaluated at time 0 (Cu dosing) all fall above the 0.5 difference threshold. There does appear to be correlation between a lower chl a/b ratio and Cu dosing with Tray 3, with almost all below 1 in Figure 7, but the broad distribution of points appeared between 0.5 and 3. The metal analysis results showed the same clear separation above a 0.7 DTW difference for samples collected after Cu dosing, but the metal content did not appear to be directly correlated with the deviation from the control in DTW. The control samples were clustered within the same region, with some deviation by Trays 2 and 3 at 24 and 48 h after dosing. However, several Tray 3 samples collected 24 h after dosing still had a high Cu content but were not observed to deviate. Therefore, it is possible to estimate that contamination is present in a given frond based on the DTW, but high Cu levels that separate for the initial dosing event do not correlate to a high DTW difference.

**Figure 8:**
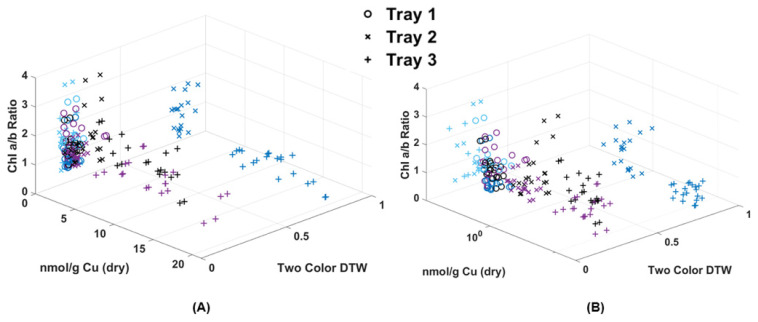
(**A**) Chl-B laser results (*x*-axis) of imaged moss fronds compared to the chlorophyll extraction results of a/b ratio (*y*-axis) and dry weight metal extraction (*z*-axis). (**B**) Shows the same data with a logarithmic scale for Cu.

Because chlorophyll and metal extraction are both destructive processes, it was not possible in this case to analyze the same fronds for both chlorophyll and Cu content. However, an attempt was made in Figure 8 to make a rough comparison using single frond results and matching Cu extraction values to the already plotted Chl a/b ratio. What resulted was a clear separation of points below and above the 0.5 two-color DTW difference. Those above 0.5 were fronds collected from Trays 2 and 3 on the same day as Cu dosing, while all other data below 0.5 were either collected before dosing (control) or after dosing (24 and 48 h later). Though the amount of Cu absorbed by the fronds was quite variable, their response using DTW was consistent. Most notable was that the shift in Chl a/b ratio to below 1 was correlated to this high initial Cu dose, while over time, the moss shifted to a higher Chl a/b ratio. In fact, the Tray 1 and Tray 2 Cu doses could prove beneficial to long-term moss health by increasing chlorophyll production. At this time, DTW applied to image analysis appears to be valuable for its ability to detect Cu contamination within moss frond samples shortly after a pollution event, but is less valuable after 24 h. Though the degree of DTW cannot specify the level of contamination, it can indicate a minimum threshold and range of contamination above the background, since no Tray 1 samples deviated through the experiment. DTW was also able to help document the correlation with the shift in chlorophyll content due to the introduction of metal.

### 2.4. Optimal Imaging Methods Applied to Chlorophyll Extraction Fluid

**Figure 9:**
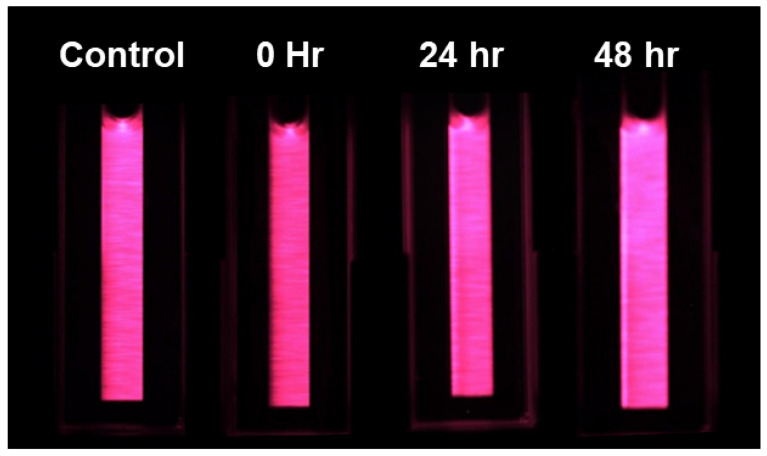
LIF images of the chlorophyll extracts in a 1 mL cuvette showing samples collected from the control, 0 h, 24 h, and 48 h after dosing.

**Figure 10:**
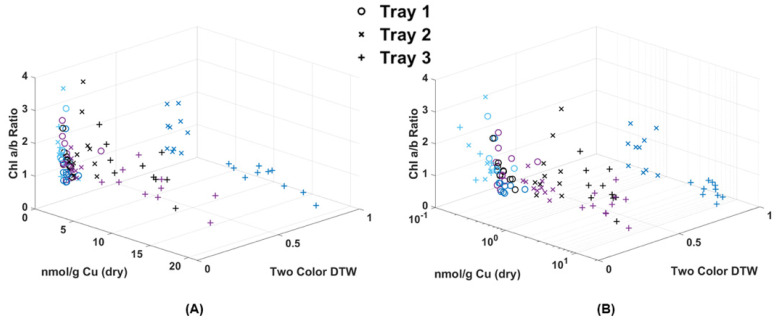
(**A**) Chl-B laser results (*x*-axis) of 1 mL cuvettes with chlorophyll solution compared to the chlorophyll extraction results of a/b ratio (*y*-axis) and dry weight metal extraction (*z*-axis). (**B**) Shows the same data with a logarithmic scale for Cu.

LIF images were also collected of the chlorophyll extraction solution in a 1 mL cuvette (Figure 9). A manual thresholding of 10 DCV was applied to the images to remove the black oxidized aluminum background, and then two-color DTW was applied. Again, because different fronds were used for metal extraction, Figure 10 is only an approximation effort to compare the Cu content to the measured chlorophyll extraction via LIF and a spectrophotometer. When reviewing Figure 10, the LIF analysis of the chlorophyll extracts shows that images taken at time 0 (Cu dosing) had the largest response in chlorophyll a/b ratio change. By 24 h after dosing, the samples became more difficult to differentiate from the control. The control samples all clustered below a 0.2 DTW two-color analysis threshold, creating a clear delineation between those during the time of dosing. When compared to Figure 8, the results were less clearly distributed between the time of dosing and sample collected at the 24- and 48-h mark. The conclusion is that the LIF of fronds is more effective than imaging the more laborious chlorophyll extract. Again, DTW cannot be used to specify the level of contamination in a sample, but it can be used to set a minimum threshold above the background.

## 3. Discussion

Previous work applied the image analysis of LIF images strictly to moss mats that filled the full view of the CMOS camera utilized in both the CoCoBi and Chl-SL. Though the CoCoBi has been successful in determining the presence of Pb and delineating the moss reaction to metal versus environmental stressors, its application toward other metals such as Cu and Zn has been inconsistent with the level of metal dosing. Thus, the Chl-SL system was developed for testing whether more chlorophyll specific lasers such as the Chl-A (445 nm) and Chl-B (462 nm) used in this work would prove to be better at detecting Cu contamination above the environmental background. Techniques for single- and two-color image analysis were also applied here to moss fronds to aid in the determination of whether moss mat techniques could be used for the targeted sampling of moss fronds that would be representative of the identified contamination and aid in complementing more traditional chemical analyses through chlorophyll and metal extraction.

To achieve image analysis, the preprocessing of images was necessary to remove the dominant pixel counts at low decimal code values (DCV), which were a result of the black aluminum oxide sheets used as a non-reflective background during the experiment. Several manual and auto thresholding levels were applied to the images and then evaluated by single-color analysis for their effective removal without a loss of valuable fluorescence information from the frond samples. It was determined that thresholding and the removal of the first 10 DCV vastly improved the results. Though auto thresholding shows promise, it was ultimately far too computationally demanding when applied to multiple color channels for the potentially minimal benefit of the better sample identification of Cu dosing. Using a grayscale version of the image for thresholding is not as computational demanding, but limits the use of multiple color channels for evaluation without any improved results for Cu dosing identification.

When comparing the different laser systems, the Chl-SL performed better than the CoCoBi whether using the Chl-A or Chl-B laser. Density difference performed poorly regardless of laser or filter system in use, but both single- and two-color DTW performed well. Use of the Chl-B filter with either Chl laser provided a better separation of frond samples from time 0 at Cu dosing than without a filter or with the Chl-A filter. This is most likely because it was best at capturing the maximum absorption of the Chl-B reaction to metal stress while also overlapping with the more dominant Chl-A absorption and emission region [30]. Though both lasers performed well, the Chl-B laser may offer the potential to separate out not only an event of metal contamination above the background levels, but also distinguish between individual levels of contamination. Whether applied to single fronds or pairs of fronds, the lasers, when fitted with the Chl-B filter and with images preprocessed to remove the first 10 DCV, were effective at detecting fronds immediately after Cu dosing using single- or two-color DTW analysis.

The evaluation of the chlorophyll and metal analysis when compared to the DTW results for both Chl lasers showed the distribution of fronds in two sections. In the first section were those DTW images of fronds that deviated from the control at the time of Cu dosing, and the second were all other samples. In the case of the chlorophyll a/b ratio, a DTW difference above 0.5 correlated to the initial Cu dose, with Tray 2 samples below an a/b ratio of 2. The Tray 3 samples with the higher dose of 100 nmol/cm^2^ fell below an a/b ratio of 1. This would indicate that the initial Cu dose may result in moss fronds responding with either a decrease in chl-a, an increase in chl-b, or both. It can be noted that a lower a/b ratio (2 or lower) can be expected in images collected that produce a DTW difference above 0.5. However, a lower a/b ratio was still present in the uncontaminated samples, and thus a separation of samples based solely on chlorophyll a/b ratio was not possible. Similarly, metal analysis revealed the separation of Trays 2 and 3 from all other samples based on their Cu levels, but only those samples collected on the Cu dosing day deviated in DTW above 0.5. Tray 3 values at 24 and 48 h after dosing still had high levels of Cu content and still did not deviate from the control when observing the DTW analysis of images. Thus, DTW deviation can help to determine a threshold of Cu contamination soon after an event, but will not detect the metal contamination already present in the plant.

It is considered a success that techniques previously only applied to moss mats have been adapted and proven here to also be applicable to individual fronds. It is believed that single- or two-color DTW analysis can be used for the analysis of any fluorescence response to aid in the evaluation and determination of difference from a control sample. Moreover, the removal of the first 10 DCV for RGB images appears as useful, if not more than, automatic thresholding, with the benefit of being computationally much faster to complete. The ability to test the manual thresholding on single fronds and validate its effectiveness when applied to pairs of fronds provides us with confidence that the technique can be used for any sample where the background may contribute a dominant number of pixels. More testing is needed for its application to backgrounds that are not uniform or may have several samples at different distances within the camera view. It is believed that automatic thresholding, contouring, and masking could aid with such datasets, but must be improved before feasibly applied for a reasonable evaluation time. If information for multiple colors is not needed or desired, automatic thresholding should be quite useful and efficient if the provided image is in grayscale.

Future work would like to further validate the results in this research and also explore the response of moss to other metals of interest with the new Chl-SL system. Application to other plant types, specifically aquatic or vascular plants, is of great interest to understand the differences in fluorescence signature between species. The method is applicable to other, widely distributed species, which will be relevant to monitor air pollution and the impact on plant physiology on a large scale. It could also be of interest to explore whether the specific levels of chl-a and chl-b can be determined within a given image collected with LIF. At this time, the image processing technique have proven useful in validating that LIF can be used to identify Cu contamination shortly after an event without confusing it for background level or historic events. The technique, with limited modification through image preprocessing, can be used on either moss mats or moss fronds with valuable results.

## 4. Materials and Methods

This work adapted the methods developed in previous studies [39,40] on moss mats and applied them to individual moss fronds that were collected, imaged, and used for the chemical analysis of chlorophyll and Cu content. The research was divided into four parts, with the first focused on the laboratory treatment and cultivation of the moss mats from which individual fronds were later collected. Part two used LIF of the CoCoBi and Chl-SL to capture color images of the moss frond response to various Cu treatments. Part three details the chemical analysis procedures for chlorophyll and Cu extracted from each frond or frond pair. Finally, the LIF images of fronds are processed to quantify the moss response to the observed absorbed Cu concentrations and corresponding measured chlorophyll levels.

### 4.1. Laboratory Procedure

*Thuidium plicatile* is an endemic moss species to Hawaiʻi (Appendix A) [41] and can be found on the island of Oʻahu along the Waʻahila Ridge Trail and State Recreational Area (21.307°, −157.797°). This moss has been consistently used in previous work [39,40] for its known response to LIF and its origin from an uncontaminated environment along the southeastern part of the Koʻolau Mountains. Moss mats were collected, taken to the lab, and rinsed and cleaned of forest litter before being divided onto three trays, each covering an area of 587 cm^2^ (7 in. × 13 in.). The trays were then placed within a laboratory grow tent that was maintained at an average temperature of 18–20 °C, 50–60% relative humidity, and 14–17 W/m^2^ ambient light (1400–1800 lux), with a day length of 10 h. An acclimatization period of two weeks was allotted before beginning the tests.

The experiment was conducted over 72 h, allowing for the collection of moss fronds from each tray to be imaged every 24 h (four samplings in total). Previous work [39] showed the reduced impact of metal toxicity on LIF detection after 24 h due to absorption by the moss species. A wire grid was constructed to divide the moss trays into 10 equal partitions from which frond samples were selected for imaging, and later chemical analysis. The first 24-h frond sampling was used to record the normal control response for each tray before the Cu treatments. At the 24-h mark (or time 0), each tray received a single dose of Cu, with Tray 1 receiving 1 nmol/cm^2^, Tray 2 10 nmol/cm^2^, and Tray 3 100 nmol/cm^2^. Fronds were collected within 10 min of Cu dosing, and chemical analysis began within 30 min of Cu dosing. Frond collection, imaging, and chemical analysis were repeated for the next two 24-h intervals (24 h and 48 h after Cu dosing). A single frond and pair of fronds were collected from each of the 10 partitions on each tray of moss. The single frond was imaged and then underwent chlorophyll extraction (2.3.1). The pair of fronds underwent the sequential elution technique (SET) for metal extraction (2.3.2). Once fronds were imaged, the trial tray was returned to the grow tent, accounting for no more than half an hour of time outside the grow tent.

### 4.2. Laser Systems and LIF Imaging Technique

Two laser systems were used to record the LIF response in the moss fronds. Both systems have been tested to evaluate the LIF in moss mats, but neither have been utilized for documenting the moss frond response. The CoCoBi [38] and a newly designed chlorophyll specific laser system (Chl-SL) both use the same CMOS camera, which can be integrated with the Baumer Camera Explorer software (v3.2.1), allowing the user to control the camera settings and capture LIF images for later analysis. The CoCoBi is a pulsed Nd:YGa dual laser system (green 532 nm laser and UV 355 nm laser) fired at a nanosecond rate (112 ns). The Chl-SL system uses two semi-conductor diode lasers at the 445 nm and 462 nm wavelengths, which are continuous. The CoCoBi has integrated time synchronized pulses that allows it to image at any time of the day, while the new system prototype does not have this feature. In order to keep the methods for comparison even between the two units, imaging was only conducted in the dark.

**Figure 11:**
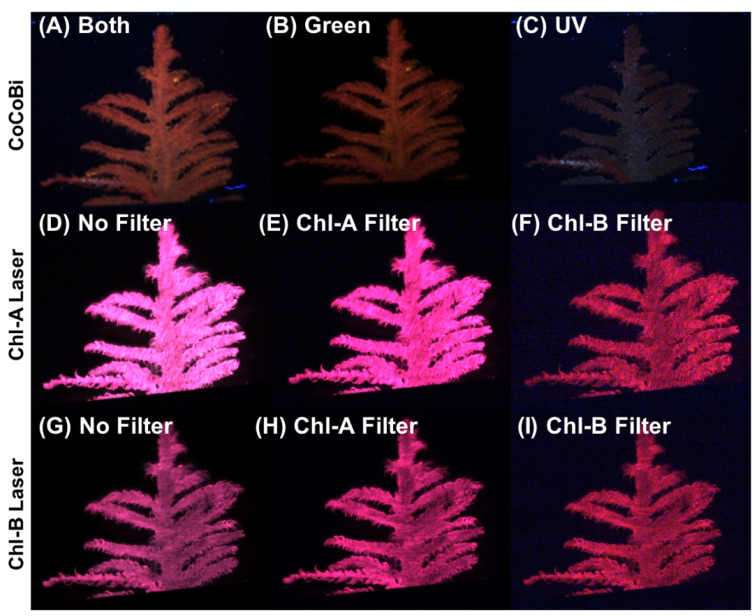
(**A**) CoCoBi using both lasers. (**B**) Only the green 532 nm CoCoBi laser. (**C**) The CoCoBi 355 nm laser. (**D**) Chl-A 445 nm laser without a filter. (**E**) Chl-A laser with 670 nm Chl-A bandpass filter. (**F**) Chl-A laser with 650 nm Chl-B bandpass filter. (**G**) Chl-B 462 nm laser without a filter. (**H**) Chl-B laser with 670 nm Chl-A bandpass filter. (**I**) Chl-B laser with 650 nm Chl-B bandpass filter.

A diffuser was mounted on each laser system to provide uniform illumination across the surface of the moss sample. Filters can be used with the CoCoBi to limit the wavelength passing to the sample to either the green or UV laser. The Chl-SL has the option of using a Chl-A bandpass filter (670 nm) or a Chl-B bandpass filter (650 nm) to capture the emission specific to chlorophyll-a and -b. These bandpass filters heavily limit the fluorescence signatures to a 10 nm wide range of light on the spectrum that can be received by the camera sensors. By limiting the wavelengths of light that are measured, we can ensure that the received signal is due to a chlorophyll emission and not another organic reaction. Figure 11 shows images of the same moss frond collected using both laser systems with all possible filter options.

Each tray of moss was divided into 10 sections using a wire grid from which a single frond and pair of fronds were selected. Samples were placed between two glass plates taped to allow only a 2-cm wide gap to remain visible. Fronds were carefully placed between these glass plates with the tip of the sample at the top of the 2-cm window and placed over a black oxidized aluminum sheet. After imaging, the same 2-cm section was cut off for chemical analysis. A control baseline for each tray was collected on the first day of the experiment. Metal dosing occurred 24 h later (at time 0 on day 2), with repeated imaging on days 3 and 4 (24 and 48 h after dosing). Moss trays were always imaged after wet deposition and when not dosed with metal; trays were only given 50 mL of DI water. Imaging of the moss fronds was conducted every 24 h in 30-min windows in order of trial number, followed by a 30-min window for chemical analysis to begin. Imaging times were held consistently for each sample between 1 and 4 pm. Images were collected by integrating the Baumer Camera Explorer software with the CMOS cameras paired with both the CoCoBi and Chl-SL. Images were collected using only the 532 nm green laser, only the 355 nm UV laser, and both lasers in tandem for the CoCoBi. The Chl-SL collected images using the 445 nm laser with no filter, 445 nm with the 650 nm filter, 445 nm with the 670 nm filter, 462 nm laser with no filter, 462 nm with the 650 nm filter, and the 462 nm with the 670 nm filter. The 650 nm band pass filter corresponds to the maximum emission peak for chlorophyll-b, while the 670 nm band pass filter corresponds to chlorophyll-a [30]. These various combinations of lasers and filters are outlined in Table 3. Preliminary testing was conducted to determine the optimal camera settings to streamline sampling to a single image per laser and/or filter combination, limiting each of the 10 collected sample areas on a tray to having nine images each (Figure 11).

### 4.3. Chemical Analysis

To assess the LIF results, the Cu uptake and changes in chlorophyll were measured using traditional chemical analysis. Chlorophyll and metals were extracted from moss fronds every 24 h immediately after laser imaging throughout the experimentation duration (starting at time 0). Metal concentrations absorbed by the plants were measured after using the sequential elution technique [42], followed by ICP-MS analysis (ICP–MS, Thermo-Fisher Element 2, University of Southern Mississippi Center for Trace Analysis). Chlorophyll extraction of a single frond [43] was followed by spectrophotometry (Hewlitt Packard Diode Array Spectrophotometer), allowing for the measurement of Chl-a and -b [44].

#### 4.3.1. Sequential Elution Technique (SET)

At the start of the experiment, 10 pairs of fronds were selected from each of the three moss trays corresponding to one of the ten grid sections. After imaging, the top 2 cm from the tip of the fronds was cut, weighed, and leached using the sequential elution technique (SET) to extract metal from the surface of the moss as well as its extra- and intracellularly bound Cu content [45,46]. Frond pairs were shaken in 10 mL of DI for 30 s to remove any unbound metals. The fronds were then removed, dried, and immersed in 10 mL of 10 mM ethylenediaminetetraacetic acid (EDTA) solution [42]. Fronds were submerged and shaken in EDTA solution for 45 min, followed by another 30 min in a fresh fraction of 10 mL of EDTA. The two EDTA fractions were combined for extracellular Cu analysis. Samples were then blotted dry, weighed, and then dried in a furnace at 50 °C for 24 h before cooling for 24 h in a desiccator. The dry weight of the cooled fronds was recorded, and finally, samples were submerged in 10 mL of 1 M HNO₃ for 30 min of shaking to induce partial digestion and the release of intracellular Cu fractions.

All samples were then analyzed for copper content (DI, EDTA, and HNO3). The individual DI water, EDTA, and nitric acid fractions were analyzed for Cu concentration using a sector-field inductively coupled plasma–mass spectrometer (ICP–MS, Thermo-Fisher Element 2) at the University of Southern Mississippi Center for Trace Analysis (CETA). A self-aspirating nebulizer (Elemental Scientific, Omaha, NE, USA) with low-flow (100 μL/min) and a Teflon spray chamber was used. Cu-63 was determined in medium resolution, and calibration was conducted using external standards made in 0.16 M ultrapure nitric acid. These were then checked against standard reference waters from the U.S. Geological Survey. There was also an in-house consistency standard measured to ensure a sensitivity check, long-term stability, and instrumental drift correction. Cu analysis was also conducted for solution blanks of DI, EDTA, and HNO₃ to determine the baseline Cu concentrations.

#### 4.3.2. Chlorophyll Extraction Method

The protocol used for chlorophyll extraction in water lettuce was adapted to determine the chlorophyll content for moss samples [47,48,49]. Ten individual fronds were collected from the 10 grided areas on each tray after imaging. Moss fronds were cut 2 cm from the tip with a sterilized razor blade before being weighed. Samples were then placed within a plastic sample tube and 2 mL of DMF (N-dimethylformamide) was added to each sample. Initial testing showed that 1 mL of DMF was needed for each cell layer of a sample, and the moss species was deemed be two cell layers thick given the volume of DMF needed for full chlorophyll extraction within 48 h [50]. DMF is more effective at limiting the continued degradation of chlorophyll than ethanol or acetone, and can be used (when kept cold) for longer periods of time after the extraction is initially conducted [43,48].

Samples were then capped, wrapped in aluminum foil, and immediately placed within a cooler with ice packs to limit the amount of light exposure before finally being placed within a freezer. After 48 h, each sample was measured by spectrophotometry for chl-a and -b using a 1 mL cuvette. The cuvette was rinsed with DMF before 1 mL of DMF was used to calibrate the spectrophotometer (Hewlitt Packard Diode Array Spectrophotometer). The measurement of samples was then conducted with cuvette cleaning and recalibration between every 30 samples. In total, 120 samples were analyzed using the extinction (wavelength of maximum light absorption) values of E663.8 and E646.8 collected for each frond and used to determine Chl-a (Equation (1)), Chl-b (Equation (2)), and Chl a + b (Equation (3)) at μg/mL levels [48]. These were then adjusted for per mg wet weight of the original frond.

(1) $$\left[Chl\;a\right]=12.00\;E^{663.8}-3.11\;E^{646.8}$$

(2) $$\left[Chl\;b\right]=20.78\;E^{646.8}-4.88\;E^{663.8}$$

(3) $$\left[Chl\;a+b\right]=17.67\;E^{646.8}+7.12\;E^{663.8}$$

### 4.4. Data Analysis

#### 4.4.1. Image Preprocessing

Previous work with moss masses [39,40] required minimal to no preprocessing of the images collected using LIF due to the sample filling the full view of the CMOS camera. Moss fronds, however, were collected with a black background, creating a large number of decimal code values at or near 0 (black). To assess the LIF response of a given frond and compare it to other frond samples, it is required that only the pixels associated with moss be considered. Two approaches were taken to determine both their effectiveness and computational demand. The first method explored applies manual thresholding, which removes decimal code values (DCV) at intervals of 5, starting at 0 and increasing to 10% (25) of the available decimal code values (256), regardless of color channel.

The second method applies automatic thresholding, which can either be applied to each color channel or can be applied to a grayscale version of the original LIF image. Previous work has seen great benefit in working with color images and comparing the plant response in all three color channels (R,G,B). Single color or grayscale histograms of an image work best with this method to evaluate for variances in the foreground and background pixels based on DCV [51,52,53]. The variance within the foreground and background pixels helps to establish a threshold of high and low pixel values within the image. Once this threshold is found, the image can be shifted to binary, where all pixels below the threshold are given a value of 0, and pixels above the threshold are given a 1. As such, pixels classified at 0 can be removed from the dataset, while those with a 1 are retained [53].

**Figure 12:**
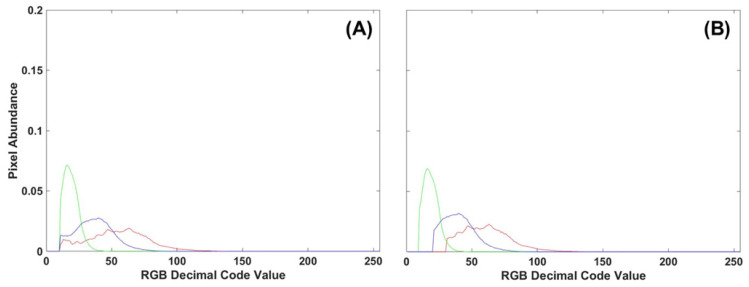
(**A**) RGB histogram of a control frond with manual thresholding applied to the first 10 DCV. (**B**) Automatic thresholding applied to the same control frond RGB histogram.

To evaluate the benefits of using either manual or automatic thresholding in the preprocessing of frond images, 10 control images from Tray 3 were compared to all 30 control images of fronds collected from all three trays. Of the manual thresholding levels explored (5, 10, 15, 20, 25), the removal of the first 10 DCV proved to have comparable results between single-color image analysis techniques (density difference or DTW). Manual thresholding at 10 DCV performed second best only to automatic thresholding. However, automatic thresholding proved computationally demanding, taking 6–10 times longer to process the same number of images with minimal improvement. A comparison of thresholding methods on RGB image histograms can be seen in Figure 12, where the manual method cuts all color channels off at 10 DCV while the automatic thresholding adjusts for each color channel. Due to decreased processing time, minimal difference to automatic thresholding, and consistent results between image analysis methods, a manual threshold of 10 DCV was applied to all frond images before single- or multi-color analysis.

#### 4.4.2. Single-Color Comparison

After preprocessing, the analysis of LIF in moss fronds was undertaken by extracting RGB (red, green, and blue) pixels from each image to create density histograms based on the decimal code value for each color channel. These histograms were then normalized using the total pixel count to create percent abundance curves. These curves were then used to calculate the difference between the treated samples and the control by either using the density difference method:

(4) $$Difference=1-\left(\sum min\left|trial\left(x\right),control\left(y\right)\right|\right)\;$$

where *x* represents the color intensities for the corresponding trial, and *y* represents the same for the control images [54], or, the difference can be calculated using dynamic time warping to fit one curve to another (DTW):

(5) $$D\left(i,j\right)=\left|x\left(i\right)-y\left(j\right)\right|+min\left\{\begin{array}{c} D\left(i+1,j\right)\\ D(i+1,j+1\\ D\left(i,j+1\right) \end{array}\right.)$$

where *x* and y represent strings of data and *i* and j represent the length of each string so that *D*(*i*,*j*) equals the best alignment distance between all data points along the lengths of *x* and *y* [55]. Sample images of the moss fronds were compared to the control images collected for each tray, 8, 16, and 24 h before Cu dosing. These were included in the results as negative values before time 0 (dosing).

#### 4.4.3. Multi-Color Comparison

Both the density difference and DTW can be used to compare single-color histograms, but only DTW can be used for two-color analysis, which has been shown to improve contaminant detection and the separation of individual samples from the control [40]. All two-color combinations (RvG, GvB, RvB) were calculated for the collected images. A mean and standard deviation to 3σ were determined for the control and used to compare whether any color channel showed a significant deviation from the control’s variability, regardless of the analysis method.

## 5. Conclusions

The work compared LIF and image processing techniques previously used only on moss mats to evaluate the chlorophyll and Cu content on moss fronds with the intent to develop a method to complement more traditional laboratory measurements. Manual thresholding was necessary as an image preprocessing step by removing the first 10 decimal code values of each color channel (RGB). Both single- and two-color DTW analyses used to compare the images to the control were effective at identifying samples immediately after Cu dosing. When compared to chlorophyll extraction, the higher DTW difference of time 0 Cu-dosed fronds correlated to lower chlorophyll a/b ratios. Metal content above the dosing level of 10 nmol/cm^2^ was also disguisable at time 0 using DTW. However, high metal content and low chlorophyll a/b ratios did not correspond directly to the DTW difference. However, the DTW difference did correlate to lower chlorophyll a/b ratios and higher metal content. Future work should re-assess preprocessing techniques and apply them to other plant and leaf types to understand the various fluorescence responses. The methodology of LIF paired with DTW image analysis was validated through a comparison to chemical analysis to identify the immediate moss response of Cu dosing. As the results of identification using DTW are only tied to high metal toxicity and changes in chlorophyll, the technique could prove useful as an aid for field and laboratory work as an initial indicator of metal contamination.

## Figures and Tables

**Table 1:** Results from chlorophyll extraction. Values shown are for 10 fronds collected every 24 h from each tray including a control. The Chl-a (nm) and Chl-b (nm) measurements were taken with a spectrophotometer and calculated by using the equations detailed in Porra (2002). Each value was adjusted for by weight (mg) of a 2 cm section of frond measured before chlorophyll extraction.

Tray 1	Chl-a /mg	Chl-b /mg	Total Chl/mg	Chl a/b Ratio	Tray 2	Chl-a /mg	Chl-b /mg	Total Chl/mg	Chl a/b Ratio	Tray 3	Chl-a /mg	Chl-b /mg	Total Chl/mg	Chl a/b Ratio
**Cont 1**	4.526	1.714	6.241	1.585	**Cont 1**	3.121	1.204	4.324	1.463	**Cont 1**	2.214	0.853	3.068	0.763
**Cont 2**	4.112	1.609	5.722	1.163	**Cont 2**	4.576	1.672	6.248	1.841	**Cont 2**	2.738	0.974	3.712	1.102
**Cont 3**	4.662	1.806	6.468	2.797	**Cont 3**	2.773	1.043	3.816	0.519	**Cont 3**	5.369	2.025	7.395	2.299
**Cont 4**	3.250	1.193	4.443	1.574	**Cont 4**	7.293	2.751	10.044	3.432	**Cont 4**	5.131	1.920	7.050	1.473
**Cont 5**	3.355	1.319	4.674	1.364	**Cont 5**	2.934	1.123	4.058	1.104	**Cont 5**	3.711	1.403	5.115	1.659
**Cont 6**	2.525	1.028	3.552	1.505	**Cont 6**	3.998	1.488	5.486	1.118	**Cont 6**	9.147	3.505	12.652	2.295
**Cont 7**	4.226	1.615	5.841	2.763	**Cont 7**	3.174	1.183	4.357	0.703	**Cont 7**	2.278	0.882	3.160	1.330
**Cont 8**	5.012	1.928	6.940	1.659	**Cont 8**	4.185	1.608	5.793	0.875	**Cont 8**	3.920	1.601	5.521	1.314
**Cont 9**	4.484	1.770	6.253	1.240	**Cont 9**	3.907	1.453	5.360	1.381	**Cont 9**	2.705	0.974	3.679	1.478
**Cont 10**	4.434	1.711	6.146	2.255	**Cont 10**	7.278	2.735	10.014	5.027	**Cont 10**	1.870	0.680	2.549	0.755
**T0-1**	1.206	0.476	1.682	0.780	**T0-1**	2.273	0.926	3.199	0.638	**T0-1**	1.836	0.830	2.665	1.030
**T0-2**	2.477	0.930	3.407	0.652	**T0-2**	1.707	0.701	2.408	0.606	**T0-2**	1.615	0.650	2.265	0.574
**T0-3**	3.395	1.268	4.663	0.673	**T0-3**	3.953	1.614	5.567	0.710	**T0-3**	2.533	1.016	3.549	0.598
**T0-4**	3.682	1.641	5.324	0.896	**T0-4**	4.240	1.725	5.966	2.081	**T0-4**	2.111	0.859	2.970	0.842
**T0-5**	2.219	1.177	3.396	0.798	**T0-5**	5.225	2.129	7.354	2.191	**T0-5**	3.441	1.344	4.785	1.042
**T0-6**	1.689	0.619	2.308	0.577	**T0-6**	5.779	2.248	8.027	0.918	**T0-6**	4.022	1.509	5.531	1.026
**T0-7**	3.561	1.435	4.996	1.288	**T0-7**	2.881	1.140	4.021	0.735	**T0-7**	1.754	0.628	2.382	0.610
**T0-8**	2.339	0.943	3.282	0.889	**T0-8**	2.877	1.227	4.104	1.426	**T0-8**	2.915	1.177	4.092	0.564
**T0-9**	1.809	0.731	2.540	0.570	**T0-9**	3.632	1.554	5.186	1.359	**T0-9**	1.810	0.698	2.508	0.590
**T0-10**	3.429	1.351	4.780	2.192	**T0-10**	2.849	1.245	4.095	1.482	**T0-10**	1.999	0.745	2.744	0.533
**T24-1**	2.640	0.992	3.632	1.792	**T24-1**	3.702	1.354	5.056	1.642	**T24-1**	3.520	1.385	4.905	1.474
**T24-2**	3.615	1.332	4.947	1.144	**T24-2**	2.681	0.998	3.679	1.238	**T24-2**	5.275	1.926	7.201	1.621
**T24-3**	3.551	1.389	4.940	0.635	**T24-3**	2.152	0.784	2.936	0.995	**T24-3**	1.799	0.742	2.542	0.607
**T24-4**	2.755	1.065	3.819	0.871	**T24-4**	2.626	1.043	3.669	1.054	**T24-4**	2.161	0.792	2.952	0.964
**T24-5**	2.348	0.892	3.240	2.005	**T24-5**	2.702	0.981	3.682	1.399	**T24-5**	2.777	1.056	3.833	1.129
**T24-6**	4.465	1.756	6.221	0.636	**T24-6**	2.525	0.945	3.470	0.794	**T24-6**	1.498	0.549	2.047	1.581
**T24-7**	4.559	1.709	6.268	1.427	**T24-7**	3.311	1.188	4.499	0.971	**T24-7**	1.677	0.596	2.272	1.294
**T24-8**	6.657	2.401	9.059	2.490	**T24-8**	4.602	1.652	6.254	1.174	**T24-8**	1.712	0.668	2.380	0.954
**T24-9**	1.710	0.632	2.341	1.183	**T24-9**	4.462	1.729	6.191	0.977	**T24-9**	1.812	0.606	2.419	1.128
**T24-10**	3.957	1.480	5.437	1.186	**T24-10**	5.013	1.936	6.949	1.191	**T24-10**	1.770	0.623	2.393	1.556
**T48-1**	3.472	1.248	4.721	1.042	**T48-1**	2.278	0.842	3.120	0.790	**T48-1**	3.160	1.087	4.246	2.463
**T48-2**	3.517	1.366	4.883	1.219	**T48-2**	3.055	1.088	4.143	1.279	**T48-2**	2.013	0.680	2.693	1.346
**T48-3**	2.692	1.003	3.696	1.251	**T48-3**	4.375	1.672	6.047	0.561	**T48-3**	6.490	2.480	8.970	1.415
**T48-4**	1.996	0.752	2.748	0.987	**T48-4**	8.229	3.007	11.236	3.755	**T48-4**	3.291	1.265	4.556	1.702
**T48-5**	2.413	0.868	3.281	2.236	**T48-5**	3.986	1.475	5.461	1.201	**T48-5**	2.946	1.123	4.070	1.347
**T48-6**	2.091	0.762	2.853	0.985	**T48-6**	3.117	1.206	4.323	1.434	**T48-6**	2.559	0.867	3.426	2.726
**T48-7**	2.889	1.005	3.894	1.326	**T48-7**	3.473	1.269	4.742	1.526	**T48-7**	6.746	2.636	9.383	1.876
**T48-8**	3.817	1.434	5.251	1.499	**T48-8**	4.453	1.666	6.119	0.964	**T48-8**	3.498	1.307	4.806	0.801
**T48-9**	2.055	0.783	2.839	1.133	**T48-9**	3.218	1.168	4.386	2.727	**T48-9**	2.642	1.082	3.724	2.134
**T48-10**	0.733	0.262	0.995	0.681	**T48-10**	5.311	1.919	7.231	1.523	**T48-10**	1.785	0.644	2.429	1.651

**Table 2:** Results from metal extraction. Values shown are of the wet and dry weight of pairs of fronds. Ten sets of fronds for each tray were collected and Cu extracted using SET every 24 h. Values were adjusted to account for the weight (g) of moss pairs and are shown in nmol/g and mg/kg levels.

Tray 1	ww	dw	Tray 2	ww	dw	Tray 3	ww	dw
**Control 1**	0.135	0.657	**Control 1**	0.073	0.270	**Control 1**	0.027	0.169
**Control 2**	0.047	0.232	**Control 2**	0.028	0.147	**Control 2**	0.043	0.261
**Control 3**	0.049	0.215	**Control 3**	0.023	0.129	**Control 3**	0.040	0.107
**Control 4**	0.058	0.258	**Control 4**	0.037	0.218	**Control 4**	0.042	0.290
**Control 5**	0.050	0.358	**Control 5**	0.034	0.233	**Control 5**	0.030	0.201
**Time 0-1**	0.090	0.304	**Time 0-1**	0.210	1.524	**Time 0-1**	2.124	14.470
**Time 0-2**	0.113	0.409	**Time 0-2**	0.261	2.136	**Time 0-2**	2.258	11.109
**Time 0-3**	0.126	0.524	**Time 0-3**	0.099	0.965	**Time 0-3**	1.351	11.039
**Time 0-4**	0.079	0.352	**Time 0-4**	0.176	1.061	**Time 0-4**	2.106	13.178
**Time 0-5**	0.098	0.387	**Time 0-5**	0.286	2.420	**Time 0-5**	1.593	12.760
**Time 0-6**	0.084	0.390	**Time 0-6**	0.069	0.434	**Time 0-6**	1.613	10.272
**Time 0-7**	0.060	0.346	**Time 0-7**	0.180	1.648	**Time 0-7**	2.065	15.628
**Time 0-8**	0.185	0.480	**Time 0-8**	0.133	1.094	**Time 0-8**	0.989	8.377
**Time 0-9**	0.059	0.334	**Time 0-9**	0.121	1.195	**Time 0-9**	2.396	18.085
**Time 0-10**	0.108	0.275	**Time 0-10**	0.195	1.578	**Time 0-10**	2.426	21.176
**Time 24-1**	0.135	0.531	**Time 24-1**	0.173	0.790	**Time 24-1**	2.047	12.101
**Time 24-2**	0.083	0.387	**Time 24-2**	0.184	0.891	**Time 24-2**	0.894	6.601
**Time 24-3**	0.068	0.331	**Time 24-3**	0.122	0.965	**Time 24-3**	3.789	18.376
**Time 24-4**	0.059	0.337	**Time 24-4**	0.207	1.541	**Time 24-4**	1.480	10.655
**Time 24-5**	0.095	0.355	**Time 24-5**	0.158	1.458	**Time 24-5**	1.495	7.566
**Time 24-6**	0.032	0.251	**Time 24-6**	0.426	1.659	**Time 24-6**	2.727	19.670
**Time 24-7**	0.063	0.279	**Time 24-7**	0.199	1.103	**Time 24-7**	2.184	12.659
**Time 24-8**	0.067	0.356	**Time 24-8**	0.236	1.400	**Time 24-8**	0.696	5.298
**Time 24-9**	0.050	0.367	**Time 24-9**	0.170	1.370	**Time 24-9**	2.138	13.261
**Time 24-10**	0.088	0.399	**Time 24-10**	0.101	0.753	**Time 24-10**	1.254	8.152
**Time 48-1**	0.091	0.457	**Time 48-1**	0.181	1.022	**Time 48-1**	2.285	13.269
**Time 48-2**	0.101	0.358	**Time 48-2**	0.233	1.148	**Time 48-2**	0.983	6.900
**Time 48-3**	0.058	0.361	**Time 48-3**	0.188	1.114	**Time 48-3**	2.035	11.327
**Time 48-4**	0.056	0.276	**Time 48-4**	0.440	2.323	**Time 48-4**	1.148	6.357
**Time 48-5**	0.089	0.289	**Time 48-5**	0.214	1.199	**Time 48-5**	1.719	10.314
**Time 48-6**	0.127	0.408	**Time 48-6**	0.208	1.254	**Time 48-6**	1.722	5.176
**Time 48-7**	0.064	0.297	**Time 48-7**	0.257	0.760	**Time 48-7**	2.124	10.785
**Time 48-8**	0.063	0.333	**Time 48-8**	0.242	1.107	**Time 48-8**	1.814	14.474
**Time 48-9**	0.098	0.361	**Time 48-9**	0.249	1.470	**Time 48-9**	0.755	5.778
**Time 48-10**	0.124	0.457	**Time 48-10**	0.339	1.497	**Time 48-10**	1.886	13.694

**Table 3:** Outline of laser and filter combinations tested.

Laser	Filter
CoCoBi—532 nm and 355 nm	None
CoCoBi—532 nm	355 nm
CoCoBi—355 nm	532 nm
Chl-SL—A 445 nm	None
Chl-SL—A 445 nm	650—chl-b
Chl-SL—A 445 nm	670—chl-a
Chl-SL—B 462 nm	None
Chl-SL—B 462 nm	650—chl-b
Chl-SL—B 462 nm	670—chl-a

## Data Availability

The MATLAB code can be accessed via https://github.com/KTruax/LIF-chlorophyll-moss-fronds (accessed on15 June 2024). The code and images analyzed for this study can be found in the OneDrive Folder: Plants—https://1drv.ms/f/c/521333978105807a/Eg2LFxizaqlEpzzUymzpRFIB_MHc8rFWQ9zr8m9z_5HVKQ?e=wicPZI (accessed on 10 June 2024).

## Appendix A

*Thuidium plicatile* Mitt. (see also *Thuidium plicatum* Mitt.) is a moss species indigenous to Hawaiʻi [41] and the one chosen to be used for experimentation and observation. The specific specimen used was collected from Oʻahu along the Wa‘ahila Ridge Trail. A frond-like species in appearance, *Thuidium plicatile* Mitt. is similar in appearance to the invasive *Hypnum plumaeforme* Wilson [56,57], which is not recorded as being present on Oʻahu [41]. *Thuidium plicatile* Mitt., *Thuidium plicatum* Mitt., and *Thuidium plicatum* Mitt. var. *brevifolium* E.B. Bartram are more broadly recognized as synonyms of *Thuidium cymbifolium* Dozy & Molk [57,58]. *Thuidium* is a genus of moss in the family *Thuidiaceae,* so named for the fronds that look like small cedar trees with creeping, branching, and pinnate leaves. One hundred and ninety-one (191) accepted species names are known in the genus *Thuidium,* all existing in temperate to tropical climates [59]. With this knowledge, if you are encouraged to work with a moss species, you do not have to rely solely upon *Thuidium plicatile* Mitt. as a means of conducting research, as there are many other species in existence.
